# Management of superficial and deep surgical site infection: an international multidisciplinary consensus

**DOI:** 10.1007/s13304-021-01029-z

**Published:** 2021-03-26

**Authors:** Gabriele Sganga, Mohamed Baguneid, Pascal Dohmen, Evangelos J. Giamarellos-Bourboulis, Emilio Romanini, Athanassios Vozikis, Christian Eckmann

**Affiliations:** 1 Division of Emergency Surgery and Trauma, Fondazione Policlinico Universitario Agostino Gemelli IRCCS, Università Cattolica del Sacro Cuore, Rome, Italy; 2 School of Medical Sciences, University of Manchester, Manchester, UK; 3 Surgical Institute, Al Ain Hospital, Al Ain, United Arab Emirates; 4 College of Medicine and Health Sciences, UAE University, Al Ain, United Arab Emirates; 5 Department of Cardiac Surgery, Heart Center Rostock, University Medicine Rostock, Rostock, Germany; 6 Department of Cardiothoracic Surgery Faculty of Health Science, University of the Free State, Bloemfontein, South Africa; 7 4th Department of Internal Medicine, National and Kapodistrian University of Athens, Medical School, Athens, Greece; 8 RomaPro Center for Hip and Knee Arthroplasty, Polo Sanitario San Feliciano, Via Enrico De Ossò 6, Rome, Italy; 9 Laboratory of Health Economics and Management, University of Piraeus, Piraeus, Greece; 10 Department of General, Visceral and Thoracic Surgery, Klinikum Hannoversch-Muenden, Goettingen University, Göttingen, Germany

**Keywords:** Surgical site infection, Management, Antimicrobial

## Abstract

Surgical site infections represent a considerable burden for healthcare systems. To obtain a consensus on the impact and future clinical and economic needs regarding SSI management in an era of multidrug resistance. A modified Delphi method was used to obtain consensus among experts from five European countries. The Delphi questionnaire was assembled by a steering committee, verified by a panel of experts and administered to 90 experts in 8 different surgical specialities (Abdominal, Cancer, Cardiac, General surgery, Orthopaedic, Thoracic, Transplant and Vascular and three other specialities (infectious disease, internal medicine microbiology). Respondents (*n* = 52) reached consensus on 62/73 items including that resistant pathogens are an increasing matter of concern and increase both treatment complexity and the length of hospital stay. There was strong positive consensus on the cost-effectiveness of early discharge (ED) programs, improvement of quality of life with ED and association between increased length of stay and economic burden to the hospital. However, established ED protocols were not widely available in their hospitals. Respondents expressed a positive consensus on the usefulness of antibiotics that allow ED. Surgeons are aware of their responsibility in an interdisciplinary team for the treatment of SSI, and of the impact of multidrug-resistant bacteria in the context of SSI. Reducing the length of hospital stays by applying ED protocols and implementing new treatment alternatives is crucial to reduce harm to patients and costs for the hospital.

**Supplementary Information:**

The online version contains supplementary material available at 10.1007/s13304-021-01029-z.

## Introduction

Despite proper precautions, surgical site infections (SSI) remain a major challenge, comprised 18.4% of all acute care-associated infections, an incidence similar to that of hospital-acquired pneumonia or urinary tract infections [1], and result in an estimated 16,049 deaths per year [2]. The incidence of SSIs is projected to increase as an aging population and increasing obesity lead to more procedures [3].

The risk of SSI varies according to type and duration of surgery, with a higher risk in emergency and/or intra-abdominal surgery involving penetration of hollow viscera, and substantially lower risk in other types of surgery (orthopaedic, cardiothoracic, vascular, neurosurgery) [4, 5]. Immunosuppressed, obese and diabetic patients are also at higher risk [6, 7].

Causative pathogens are usually commensal bacteria associated with the surgical site [8], and thus tend to vary according to the procedure [9]. When organisms are isolated, the most frequently identified bacteria include *Staphylococcus aureus,* coagulase-negative Staphylococci,* Enterococcus* sp., and *Escherichia coli* [10, 11]. Whereas Gram-positive microorganisms like *S. aureus* are the most common causative pathogens, Gram-negative bacteria including Enterobacteriaceae may cause SSIs, especially after abdominal procedures. Another element of variability is the risk of infection with antimicrobial-resistant (AMR) bacteria. SSIs involving AMR pathogens are associated with significant increases in the length of hospitalization and costs [12].

Institutional infection surveillance reports are the most relevant source of information [13], and their existence has been shown to reduces SSI rates [14]. accurate estimation of SSI rates is hindered when onset occurs after discharge [15, 16]. Meta analysis of data from 1.4 million surgeries in 15 countries identified 141,347 SSIs, of which 84,984 (60.1%) appeared after discharge [17]. Recently, a patient-self-assessment questionnaire has been validated for assessing SSIs after hospital discharge [18].

The multidisciplinary team is responsible for stratifying SSI risk and coordinating surgical and antimicrobial approaches for treating infections [19]. Therefore, members should be familiar with local epidemiology, antimicrobial resistance, and the available antimicrobial options. Reliable data on clinicians’ perspectives on SSI management are lacking. To this end, we have conducted a modified Delphi process to develop an expert consensus on the management of superficial and deep incisional SSIs from a large panel of experts working in different specialties and European countries.

## Methods

### Delphi method

We used a modified Delphi process to obtain consensus among 52 European experts on the management of surgical site infection [20]. The process was conducted by a 7-member multinational steering committee comprising Surgeons (Cardiac, General and Emergency, Orthopaedic, Thoracic and Vascular), an Infectious Disease expert and a Health Economics expert (the Authors) assembled and led by C.E. and G.S. Each committee member prepared candidate Delphi questionnaire statements in their area of expertise based on their experience and familiarity with the literature and guidelines. These candidate statements were presented and discussed at an organisational meeting in Rome on June 14, 2019. The draft questionnaire was submitted to a round of validation by a panel of 8 experts identified by the steering committee (Listed in acknowledgements section). The validation panel provided feedback on the legibility and coherence of the statements. The final revised version contains 15 statements with a total of 73 items divided among 4 SSI topics (Epidemiology, Management, Consequences, and Antibiotic therapy approach) (Appendix I—Supplemental File 1).

In addition to the consensus items, the questionnaire contained items to survey the respondents’ level of expertise, local epidemiology and the medical setting that they work in. This ancillary information was used when interpreting the consensus items. The questionnaire asked experts to indicate their level of agreement with each item on a 5-point Likert scale, in which 1-strongly disagree, 2-disagree, 3-agree, 4-more than agree, 5-strongly agree. It was determined a priori that responses of 1–2 would be considered disagreement, whereas responses of 3–5 would be considered agreement, and that consensus would be defined as ≥ 66% agreement/disagreement. This was explained in the instructions for competing the questionnaire.

The questionnaire was distributed electronically to a purposive sample of 90 surgeons in 5 European countries (France, Germany, Greece, Italy and the UK), which was identified by the steering committee based on their contributions in the field (e.g., national/international leadership, publications, work on commissions). Participating experts expressed their opinions anonymously, in accordance with Delphi methodology. Responses were analysed and discussed by the steering committee at a second meeting in Rome on October 22, 2019.

### Definition of an SSI

For the purpose of this consensus, we have considered superficial and deep incisional infections occurring after an index surgery (Appendix 2—Supplemental File 2), according to the US Centers for Disease Control and Prevention (US CDC) definition [21, 22]. For deep incisional infections, we considered only major abscesses and deep cellulitis. Organ/space infections (US CDC classification A3) were not included in this consensus as they could be confounded by complications not related to measures for the reduction of SSI (e.g., anastomotic leakage).

## Results

### Participants

Of 90 surveys distributed, 52 experts responded (58%). Respondents were evenly distributed between Western and South-eastern Europe. Respondents were practicing in France (1), Germany (12), Greece (15), Italy (12), or United Kingdom (12), and comprised 36 surgeons from 8 major specialties and 16 infectious disease experts (Appendix III—Supplementary Table 1).

Consensus was achieved on 62 of 73 items. The remaining 11 items (15%) did not reach consensus; however, most of these were ancillary items that had been included for ascertaining the respondents’ expertise, local epidemiology and clinical setting; therefore, a consensus would not be expected/appropriate on these items. As such, they were not addressed in a further Delphi round but commented on as useful insight into possible differences among geographic areas and different surgical specialties.

### Delphi results

#### Epidemiology (Fig. 1)

**Fig. 1:**
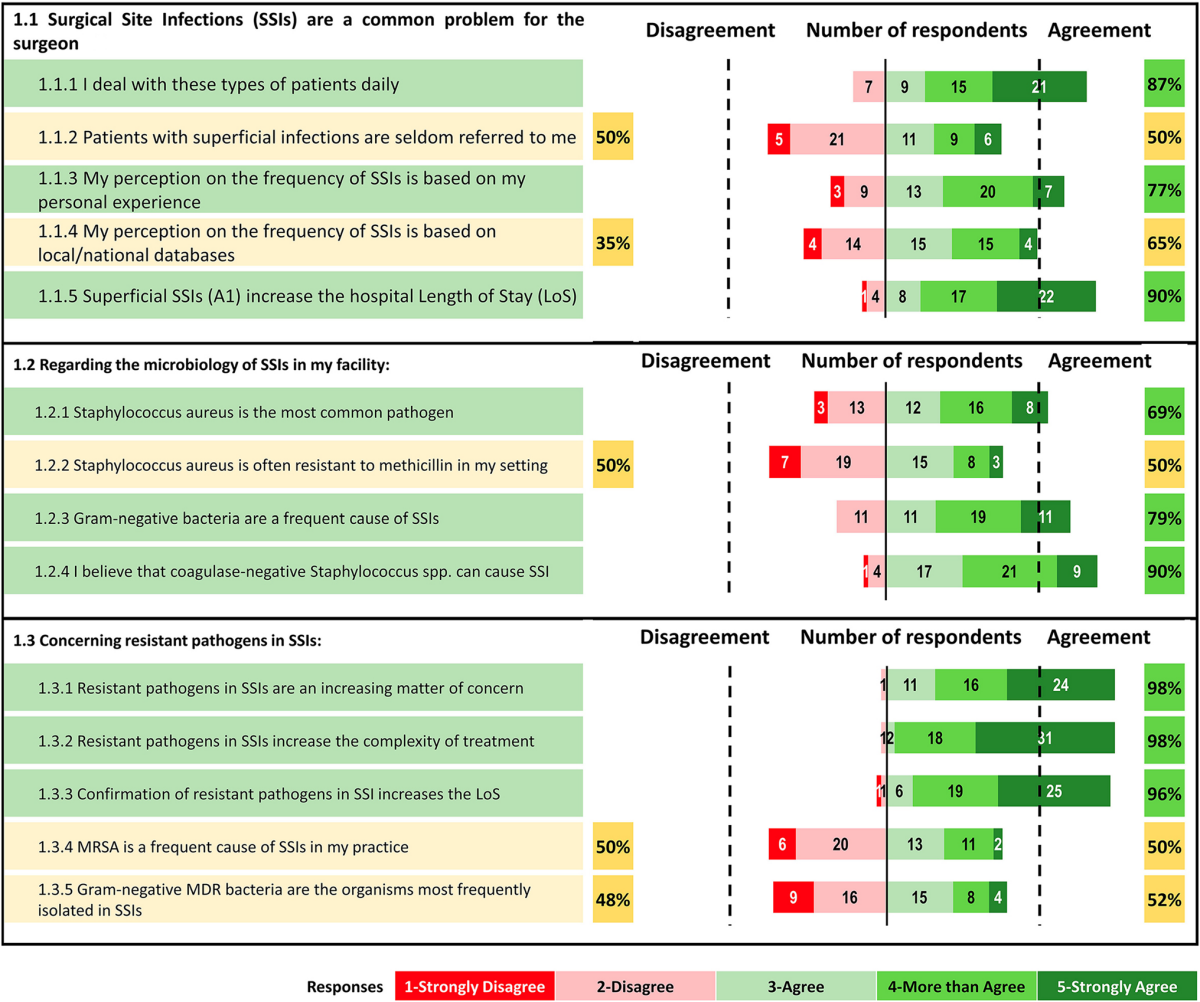
Percentage of agreement and number of respondents on each statement of the Delphi questionnaire. Topic 1: Epidemiology

##### Statement 1.1: SSI epidemiology

We sought to confirm that the experts do indeed face this problem on a regular basis, therefore, the strong agreement on item 1.1.1 indicates that they self-identity as experts in this area. They rely on both personal experience (item 1.1.3) and local/national databases as a source of information on SSI frequency (item 1.1.4); they agree also that superficial SSIs can increase LOS (item 1.1.5). Meanwhile, only half of the experts indicated that superficial SSIs are frequently referred to them (item 1.1.2).

##### Statement 1.2: SSI microbiology

Considering the microbiology of SSIs in their facilities, respondents agreed that *Staphylococcus aureus* is the most common pathogen (item 1.2.1); however only half of the respondents agreed that MRSA is frequent in their setting (item 1.2.2). There was agreement that Gram-negative pathogens were frequent causes of SSIs (item 1.2.3), and that coagulase-negative *Staphylococcus* spp. can cause SSIs (item 1.2.4).

##### Statement 1.3: Antimicrobial resistance in SSI

Respondents strongly agree that resistant pathogens are an increasing matter of concern in SSIs (item 1.3.1), that they increase both treatment complexity (item 1.3.2), and LOS (item 1.3.3). With respect to their own practices, only half of the experts indicated that MRSA is a frequent cause of SSIs (item 1.3.4); whereas 52% considered Gram-negative MDR bacteria to be the organisms most frequently isolated in SSIs (item 1.3.5).

#### Management (Fig. 2)

**Fig. 2:**
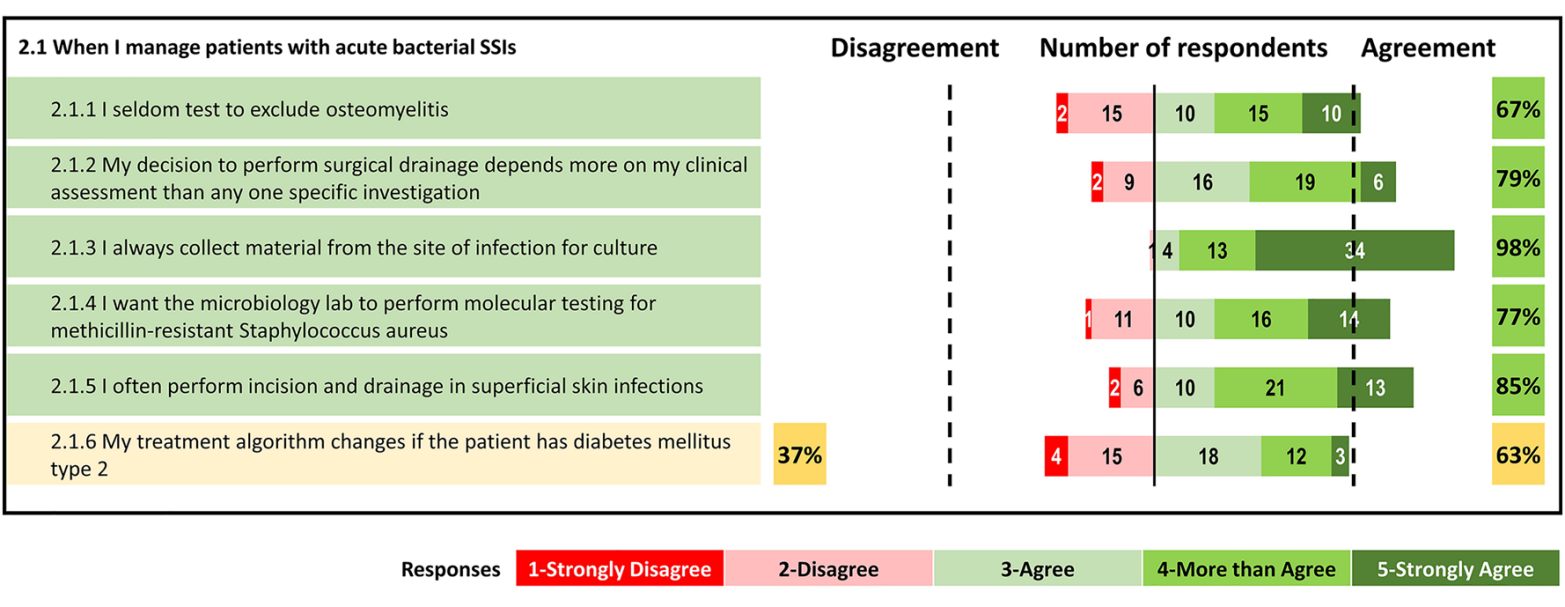
Percentage of agreement and number of respondents on each statement of the Delphi questionnaire. Topic 2: Management

##### Statement 2: Main principles of management

Regarding the management of patients with acute bacterial SSIs, there was a weak positive consensus that testing should be performed to exclude osteomyelitis (item 2.1.1). Respondents agreed that the decision to perform surgical drainage should be based mainly on clinical assessment (item 2.1.2); there was also agreement on the need to collect material from an infected wound for culture (item 2.1.3) and on requesting molecular testing for MRSA (item 2.1.4); also, most experts agreed on the need to perform incision and drainage in superficial skin infections (item 2.1.5). There was no consensus on the need to modify the treatment algorithm for patients with diabetes mellitus type 2 (item 2.1.6).

#### Consequences (Fig. 3)

**Fig. 3:**
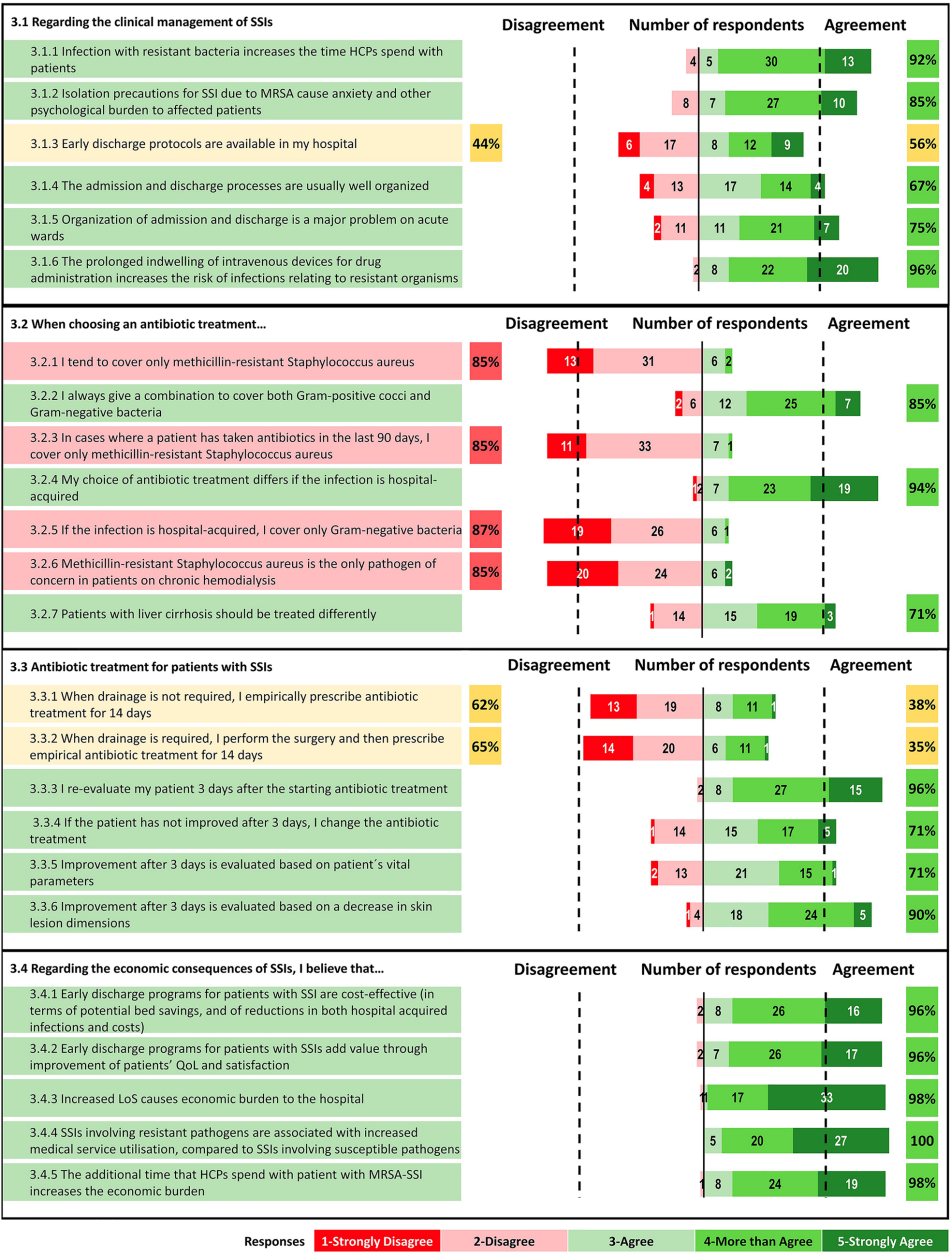
Percentage of agreement and number of respondents on each statement of the Delphi questionnaire. Topic 3: Consequences

##### Statement 3.1: Clinical management of SSI

Respondents strongly agree that infection with resistant bacteria increases the time that Health Care Professionals spend with patients (item 3.1.1) and that isolation precautions for SSIs due to MRSA cause anxiety and additional psychological burden for affected patients (item 3.1.2). Only 56% confirmed that ED protocols are available in their hospitals (item 3.1.3); however, there was agreement that hospital admissions and discharges are usually well organized (item 3.1.4), while the organization of admission and discharge in acute wards is a major problem (item 3.1.5). There was strong agreement that prolonged indwelling intravenous devices for drug administration increase the risk of infections with resistant organisms (item 3.1.6).

##### Statement 3.2: Choice of antibiotics

The group consensus was consistently negative on the four items concerning the use of narrow-spectrum antimicrobials: covering only MRSA (item 3.2.1); covering only MRSA in patients who have taken antibiotics in the last 90 days (item 3.2.3), covering only Gram-negative bacteria in hospital-acquired infections (item 3.2.5), and covering only MRSA in patients on chronic haemodialysis (item 3.2.6). On the other hand, respondents agreed on the need to administer regimens that cover both Gram-positive cocci and Gram-negative bacteria (item 3.2.2), and that the antimicrobial treatment choice differs if the infection is hospital-acquired (3.2.4) and if patient has liver cirrhosis (item 3.2.7).

##### Statement 3.3: Antibiotic treatment for patients with SSIs

There was no consensus on whether empirical antibiotic treatment should be prescribed for 14 days, irrespective of whether drainage is required (items 3.3.1 and 3.3.2). There was positive consensus on the four items dealing with wound assessment 3 days after starting antibiotic treatment: re-evaluation after 3 days (3.3.3), changing the antibiotic treatment if there is no improvement (3.3.4), evaluating response to treatment based on the patient’s vital parameters (3.3.5), and evaluation based on a decrease in skin lesion dimensions (3.3.6).

##### Statement 3.4: Regarding the economic consequences of SSI

There was strong positive consensus on all five items concerning the economic consequences of SSI: cost-effectiveness of ED programs (3.4.1), improvement of quality of life with ED (3.4.2), association between increased length of stay and economic burden to the hospital (3.4.3), increased service utilization for SSIs caused by resistant microorganisms, (3.4.4), and increased healthcare professional workload when SSIs involve MRSA (3.4.5).

#### Antibiotic therapy approach (Fig. 4)

**Fig. 4:**
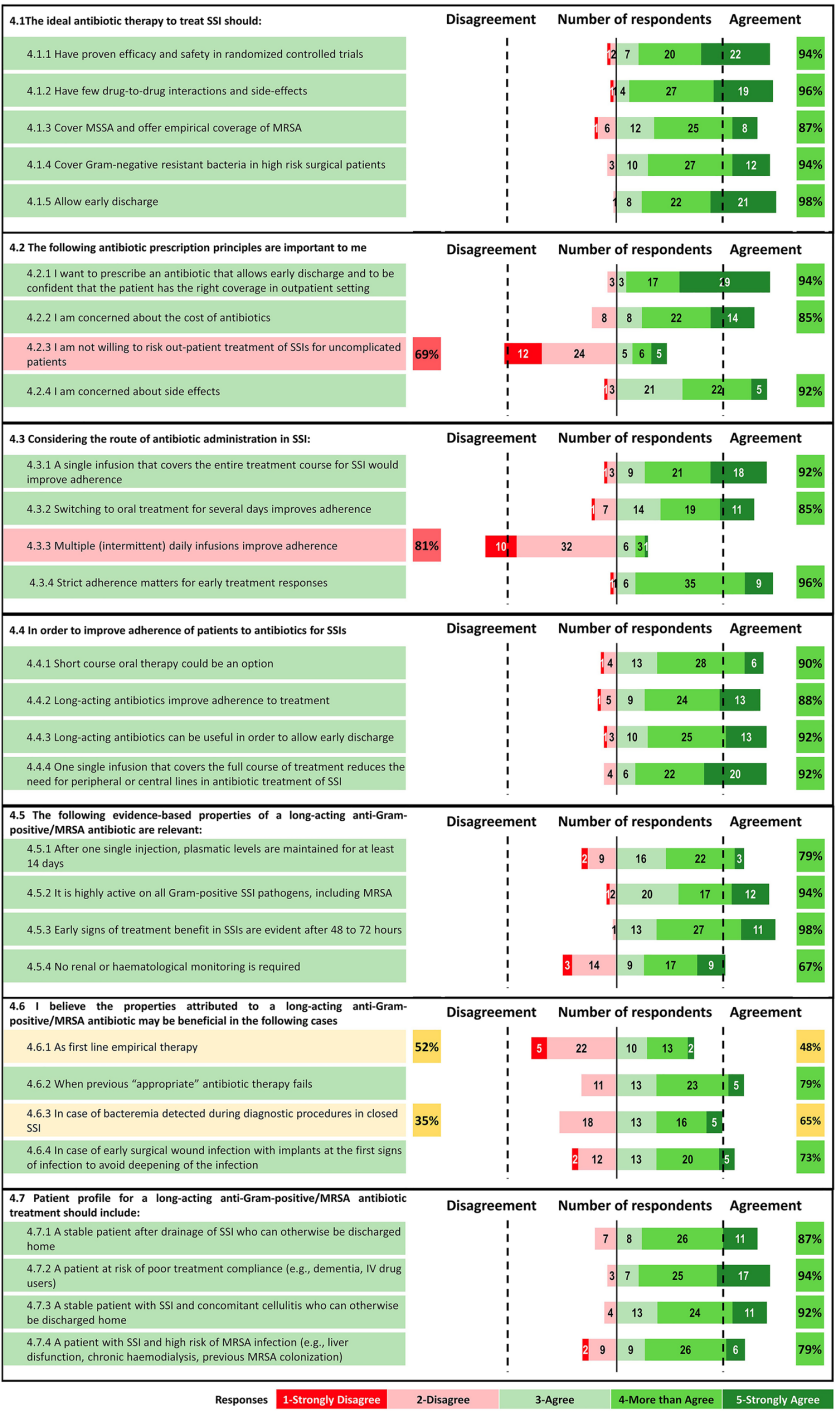
Percentage of agreement and number of respondents on each statement of the Delphi questionnaire. Topic 4: Antibiotic therapy approach

##### Statement 4.1: Ideas on the ideal antibiotic therapy

Considering the ideal antimicrobial therapy to treat SSIs, respondents strongly agreed on the following characteristics: efficacy and safety established in randomized controlled trials (4.1.1), few drug-to-drug interactions and side-effects (4.1.2); coverage of MSSA and offer empirical coverage of MRSA (4.1.3), and coverage of Gram-negative resistant bacteria in high-risk surgical patients (4.1.4). These characteristics are linked with ED (4.1.5).

##### Statement 4.2: Prescription principles

When considering important antibiotic prescription principles, respondents expressed a positive consensus for antibiotics that allow ED while instilling confidence that the patient has appropriate coverage in the outpatient setting. Most respondents were concerned about the cost of antibiotics (4.2.2) and about side effects (4.2.4); nearly one third of clinicians (31%) were not willing to risk outpatient treatment for SSIs in uncomplicated patients (4.2.3).

##### Statement 4.3: Route of antibiotic administration

Respondents agree that strict adherence is important for early treatment responses (4.3.4), that a single infusion covering the entire treatment course for SSI would improve adherence (4.3.1) and that switching to oral treatment for several days improves adherence (4.3.2). They did not agree that multiple (intermittent) daily infusions improve adherence (4.3.3).

##### Statement 4.4: Improving compliance

Respondents agreed that adherence to antibiotics for SSI can be improved with short-course oral therapy (4.4.1). There was a positive consensus that the use of long-acting antibiotics improves adherence (4.4.2) and allow ED (4.4.3), and that one single infusion that covers the full treatment course reduces the need for peripheral or central lines in antibiotic treatment of SSI (4.4.4).

##### Statement 4.5: Evidence-based properties of long-acting anti-Gram-positive/MRSA antibiotics

There was positive consensus that all of the following evidence-based properties of a long-acting anti-Gram-positive/MRSA antibiotic are relevant: plasma levels maintained for at least 14 days after one single injection (4.5.1); high activity on all Gram-positive SSI pathogens including MRSA (4.5.2), early signs of treatment response after 48–72 h (4.5.3) and no need for renal or haematological monitoring (4.5.4).

##### Statement 4.6: Settings for long-acting anti-Gram-positive/MRSA antibiotics

Regarding settings where long-acting anti-Gram-positive/MRSA antibiotics may be beneficial, there was no consensus for first-line empirical therapy (4.6.1), or for bacteraemia detected in closed SSIs (4.6.3). There was a positive consensus for rescue therapy when previous “appropriate” antibiotic therapies fail (4.6.2) and at the first sign of infection in surgical wounds with implants, to avoid deepening of the infection (4.6.4).

##### Statement 4.7: Patient profile for long-acting anti-Gram-positive/MRSA antibiotic treatments

There was positive consensus that patient suitable for long-acting anti-Gram-positive/MRSA antibiotic treatments should be stable after SSI drainage and otherwise candidates to be discharged home (4.7.1), patients at risk of poor treatment compliance (4.7.2), stable patient with SSI and concomitant cellulitis who can otherwise be discharged home (4.7.3) and patients with SSI at high risk of MRSA infection (4.7.4).

## Discussion

This Delphi process revealed that the participating European experts were aware of the burden of SSIs since consensus was achieved in the great majority of items. Experts do consider MRSA and resistant Gram-negative bacteria as common pathogens, but they also recognize one emerging limitation that should be taken into consideration: the financial burden that may rise from prolonged hospitalization and the need for strategies to promote ED.

Responses on epidemiology are consistent with ECDC surveillance data [23]. Respondents agreed almost unanimously on the importance of collecting samples from SSIs for microbiological analysis; three quarters of them would like their microbiology laboratories to perform molecular testing for MRSA.

As expected from a survey conducted across regions, there were different responses on the perceived frequency of MRSA in SSIs. This is consistent with surveillance data showing a mean incidence of MRSA in European countries of 16.4%, but a range from 0 to 43% [24]. About half of the respondents indicated that AMR Gram-negative bacteria are the most frequent SSI pathogens in their setting. This is consistent with results from 633 SSIs in a Greek study that found high rates of AMR associated with Gram-negative pathogens from skin infections [11].

Whereas the incidence of AMR Gram-negative infections is higher in several Southern and Eastern European countries [24, 25], it is not clear that these pathogens are implicated in a high percentage of SSIs. The response of our panel may reflect heightened awareness of the increasing burden imposed by AMR pathogens in any kind of infection. Each year in the EU and EEA alone they are estimated to cause 33,000 deaths and 874,000 disability-adjusted life years (DALYs) [26].

The lack of consensus on the duration of antimicrobial treatment reflects the ongoing debate. In general, shorter treatment durations may be safer and as effective for uncomplicated bacterial infections [27, 28]. Longer courses are appropriate for deep-seated or severe infections that require a treatment regimen with optimised pharmacokinetics and pharmacodynamics and may benefit from a treatment duration that is based on clinical response and biomarkers [29]. Recent endpoints for acute bacterial skin and skin structure infections propose early response criteria based on lesion size after 48–72 h [30]; in our survey, this is reflected in the consensus on the need to re-evaluate patients after 3 days.

These experts were aware of the substantial economic burden associated with SSIs, mainly due to increased LOS [31–33], and agreed that discharge protocols are important for dealing with this issue; however, the survey results on the availability of ED protocols (3.1.3) indicate they are used only sparsely. This confirms the results of a pan-European retrospective study in 1500 patients with complicated skin and soft-tissue infections due to MRSA conducted to identify the potential to switch from iv to oral antibiotic medication and facilitate ED. The mean length of stay in that study was almost 20 days and the potential for ED almost 40% [34]. While ED programs for appropriate patients can reduce hospital costs, they have the added advantages of reducing the risk of hospital-acquired infections [35].

There was a strong consensus that the ideal antibiotic therapy to treat SSIs should allow ED for otherwise eligible patients (4.1.5), while ensuring that they will have suitable antimicrobial coverage (4.2.1; 4.2.3). Strategies for achieving this include switching from iv to oral formulations or administering long-acting drugs that allow single shot or weekly administrations.

Compliance is a major determinant of therapeutic success [36]; however, adherence to oral antibiotic therapy after hospital discharge is suboptimal and may be associated with poor outcomes [37]. In further considerations on the route of administration, the panel expressed a consensus for using a single infusion covering the entire antimicrobial course (4.3.1) to improve adherence by avoiding the need for multiple (daily) dosing (4.3.3). This is consistent with the findings of a Delphi process conducted with 238 clinicians (61% infectious disease specialists) in 10 European countries, which showed a strong consensus on the importance of adherence and the advantages of administering long-acting drugs for acute bacterial skin and skin structures infections [38]. Long-acting lipoglycopeptide antimicrobials such as dalbavancin and oritavancin provide intrinsic treatment adherence due to their long half-lives [39].

The panel did not reach a consensus on the appropriateness of long-acting agents in the first-line empirical setting (4.6.1). This is consistent with the concerns regarding Gram-negative pathogens and preference for broad-spectrum coverage expressed in items (3.2). However, there was strong agreement that long-acting agents would be beneficial as a rescue treatment when a previous appropriate antimicrobial treatment fails (4.6.2) or in the context of preventing the deepening of an SSI that is associated with an implant (4.6.4). The profile for a patient with SSI to be treated with long-acting agents was that of a stable patient, possible after SSI drainage, possibly with concomitant cellulitis, who could otherwise be discharged home (4.7.1; 4.7.3). Other characteristics could include the risk of poor compliance and/or MRSA infection (4.7.2; 4.7.4).

This investigation has several strengths: To the best of our knowledge, this is the first publication to address the important global issue of SSI burden and treatment through a Delphi method among experts in the field. Moreover, it comprises the consensus of a broad variety of surgical disciplines from five European countries. The high percentage of consensus reflects that clinicians are facing many of the same challenges, regardless of their specialty and nationality.

This study has several limitations. The Delphi process was confined to the steering committee which designed the questions and received feedback from a validation group of experts (see above). The validated questionnaire was administered to the Delphi group of international experts in the field of SSI only once. The objectivity of the results may have increased with an additional round; however, this strategy has been used successfully in other Delphi procedures with heterogeneous panels [9, 38, 40]. Secondly, the Delphi population was identified through purposive sampling based on the contributions of potential participants to the international literature and their professional reputations, with the aim of obtaining input from established experts in different specialties working in several European countries; this should be considered when interpreting the responses.

## Conclusions

This Delphi process has revealed that experts are aware of their responsibility in an interdisciplinary team for the treatment of SSI. The issue of multidrug-resistant gram-positive and gram-negative bacteria causing SSI is well recognised, as well as the implications for the affected patients and the hospital. Reducing LOS while maintaining the same quality of care is an important aim in SSI, to reduce harm to the patients and costs for the hospital. Wider availability of early discharge protocols and the implementation of new treatment alternatives like long-acting antibiotics will be crucial for the optimal future management of SSI in an era of multidrug resistance.

## Supplementary Information

Below is the link to the electronic supplementary material.

Supplementary file1 (DOCX 13 kb)

Supplementary file2 (DOCX 31 kb)

Supplementary file3 (DOCX 24 kb)

## Data Availability

“Not applicable”. Data sharing not applicable to this article as no datasets were generated or analyzed during the current study.

## Abbreviations

AMR: Antimicrobial-resistance
CI: Confidence interval
ECDC: The European Centre for Disease Prevention and Control
ED: Early discharge
LOS: Length of (hospital) stay
MRSA: Methicillin-resistant *Staphylococcus aureus*
MSSA: Methicillin-sensitive *Staphylococcus aureus*
SSI: Surgical site infection
UI: Uncertainty interval
US CDC: US Centers for Disease Control and Prevention
