# Supplementary material for: Management of superficial and deep surgical site infection: an international multidisciplinary consensus
Source: Updates Surg. 2021 Mar 26;73(4):1315–25. doi: 10.1007/s13304-021-01029-z (PMC8397635; doi:10.1007/s13304-021-01029-z)
Supplement: Supplementary file 1 — Supplementary file1 (DOCX 13 kb) [file 13304_2021_1029_MOESM1_ESM.docx]

Supplementary Table 1. The surgical specialties of the 52 respondents, and the countries where they practice.

| Country | Number |
| --- | --- |
| Italy | 12 |
| France | 1 |
| Germany | 12 |
| Greece | 15 |
| United Kingdom | 12 |
| **Surgical specialty** | **Number** |
| Abdominal | 2 |
| Cancer | 6 |
| Cardiac | 2 |
| General | 18 |
| Orthopaedic | 1 |
| Thoracic | 3 |
| Transplant | 2 |
| Vascular | 2 |
| Other specialities | 16 |
