# Supplementary material for: Management of superficial and deep surgical site infection: an international multidisciplinary consensus
Source: Updates Surg. 2021 Mar 26;73(4):1315–25. doi: 10.1007/s13304-021-01029-z (PMC8397635; doi:10.1007/s13304-021-01029-z)
Supplement: Supplementary file 2 — Supplementary file2 (DOCX 31 kb) [file 13304_2021_1029_MOESM2_ESM.docx]

**Additional File 1.** Delphi survey

**1. EPIDEMIOLOGY**

| **1.1 Surgical Site Infections (SSIs) are a common problem for the surgeon** |
| --- |
| I deal with these types of patients daily |
| Patients with superficial infections are seldom referred to me |
| My perception on the frequency of SSIs is based on my personal experience |
| My perception on the frequency of SSIs is based on local/national databases |
| Superficial SSIs (A1) increase the hospital Length of Stay (LoS) |

| **1.2 Regarding the microbiology of SSIs in my facility…** |
| --- |
| *Staphylococcus aureus* is the most common pathogen |
| *Staphylococcus aureus* is often resistant to methicillin in my setting |
| Gram-negative bacteria are a frequent cause of SSIs |
| I believe that coagulase-negative *Staphylococcus* spp. can cause SSI |

| **1.3 Concerning resistant pathogens in SSIs:** |
| --- |
| Resistant pathogens in SSIs are an increasing matter of concern |
| Resistant pathogens in SSIs increase the complexity of treatment |
| Confirmation of resistant pathogens in SSI increases the LoS |
| MRSA is a frequent cause of SSIs in my practice |
| Gram-negative MDR bacteria are the organisms most frequently isolated in SSIs |

**2. MANAGEMENT**

| **2.1 When I manage patients with acute bacterial SSIs…** |
| --- |
| I seldom test to exclude osteomyelitis |
| My decision to perform surgical drainage depends more on my clinical assessment than any one specific investigation |
| I always collect material from the site of infection for culture |
| I want the microbiology lab to perform molecular testing for methicillin-resistant *Staphylococcus aureus* |
| I often perform incision and drainage in superficial skin infections |
| My treatment algorithm changes if the patient has diabetes mellitus type 2 |

**3. CONSEQUENCES**

| **3.1 Regarding the clinical management of SSIs** |
| --- |
| Infection with resistant bacteria increases the time HCPs spend with patients |
| Isolation precautions for SSI due to MRSA cause anxiety and other psychological burden to affected patients |
| Early discharge protocols are available in my hospital |
| The admission and discharge processes are usually well organized |
| Organization of admission and discharge is a major problem on acute wards |
| The prolonged indwelling of intravenous devices for drug administration increases the risk of infections relating to resistant organisms |

| **3.2 When choosing an antibiotic treatment…** |
| --- |
| I tend to cover only methicillin-resistant *Staphylococcus aureus* |
| I always give a combination to cover both Gram-positive cocci and Gram-negative bacteria |
| In cases where a patient has taken antibiotics in the last 90 days, I cover only methicillin-resistant *Staphylococcus aureus* |
| My choice of antibiotic treatment differs if the infection is hospital-acquired |
| If the infection is hospital-acquired, I cover only Gram-negative bacteria |
| Methicillin-resistant *Staphylococcus aureus* is the only pathogen of concern in patients on chronic haemodialysis |
| Patients with liver cirrhosis should be treated differently |

| **3.3 Antibiotic treatment for patients with SSIs** |
| --- |
| When drainage is not required, I empirically prescribe antibiotic treatment for 14 days |
| When drainage is required, I perform the surgery and then prescribe empirical antibiotic treatment for 14 days |
| I re-evaluate my patient 3 days after starting the antibiotic treatment |
| If the patient has not improved after 3 days, I change the antibiotic treatment |
| Improvement after 3 days is evaluated based on patient´s vital parameters |
| Improvement after 3 days is evaluated based on a decrease in skin lesion dimensions |

| **3.4 Regarding the economic consequences of SSIs, I believe that…** |
| --- |
| Early discharge programs for patients with SSI are cost-effective (in terms of potential bed savings, and of reductions in both hospital-acquired infections and costs) |
| Early discharge programs for patients with SSIs add value through improvement of patients’ QoL and satisfaction |
| Increased LoS causes economic burden to the hospital |
| SSIs involving resistant pathogens are associated with increased medical service utilisation, compared to SSIs involving susceptible pathogens |
| The additional time that HCPs spend with patient with MRSA-SSI increases the economic burden |

**4. ANTIBIOTIC THERAPY APPROACH**

| **4.1 The ideal antibiotic therapy to treat SSI should…** |
| --- |
| Have proven efficacy and safety in randomized controlled trials |
| Have few drug-to-drug interactions and side effects |
| Cover MSSA and offer empirical coverage of MRSA |
| Cover Gram-negative resistant bacteria in high risk surgical patients |
| Allow early discharge |

| **4.2 The following antibiotic prescription principles are important to me** |
| --- |
| I want to prescribe an antibiotic that allows early discharge and to be confident that the patient has the right coverage in outpatient setting |
| I am concerned about the cost of antibiotics |
| I am not willing to risk out-patient treatment of SSIs for uncomplicated patients |
| I am concerned about side effects |

| **4.3 Considering the route of antibiotic administration in SSI:** |
| --- |
| A single infusion that covers the entire treatment course for SSI would improve adherence |
| Switching to oral treatment for several days improves adherence |
| Multiple (intermittent) daily infusions improve adherence |
| Strict adherence matters for early treatment responses |

| **4.4 In order to improve adherence of patients to antibiotics for SSIs** |
| --- |
| Short course oral therapy could be an option |
| Long-acting antibiotics improve adherence to treatment |
| Long-acting antibiotics can be useful in order to allow early discharge |
| One single infusion that covers the full course of treatment reduces the need for peripheral or central lines in antibiotic treatment of SSI |

| **4.5 The following evidence-based properties of a long-acting anti-Gram-positive/MRSA antibiotic are relevant:** |
| --- |
| After one single injection, plasma levels are maintained for at least 14 days |
| It is highly active on all Gram-positive SSI pathogens, including MRSA |
| Early signs of treatment benefit in SSIs are evident after 48 to 72 hours |
| No renal or haematological monitoring is required |

| **4.6 I believe the properties attributed to a long-acting anti-Gram-positive/MRSA antibiotic may be beneficial in the following cases:** |
| --- |
| As first line empirical therapy |
| When previous “appropriate” antibiotic therapy fails |
| In case of bacteraemia detected during diagnostic procedures in closed SSI |
| In case of early surgical wound infection with implants at the first signs of infection to avoid deepening of the infection |

| **4.7 Patient profile for a long-acting anti-Gram-positive/MRSA antibiotic treatment should include:** |
| --- |
| A stable patient after drainage of SSI who can otherwise be discharged home |
| A patient at risk of poor treatment compliance (e.g., dementia, IV drug users) |
| A stable patient with SSI and concomitant cellulitis who can otherwise be discharged home |
| A patient with SSI and high risk of MRSA infection (e.g., liver disfunction, chronic haemodialysis, previous MRSA colonization) |

**Additional file 2.** Criteria for Defining Surgical Site Infections (SSIs) based on the CDC definitions for superficial and deep incisional infections, excluding organ and body space infections.^22^

| **Superficial Incisional SSI** - Infection occurs within 30 days after the operation and infection involves only skin or subcutaneous tissue of the incision and at least one of the following:   1. Purulent drainage, with or without laboratory confirmation, from the superficial incision. 2. Organisms isolated from an aseptically obtained culture of fluid or tissue from the superficial incision. 3. At least one of the following signs or symptoms of infection: pain or tenderness, localized swelling, redness, or heat and superficial incision is deliberately opened by surgeon, unless incision is culture-negative. 4. Diagnosis of superficial incisional SSI by the surgeon or attending physician.   Do not report the following conditions as SSI:   1. Stitch abscess (minimal inflammation and discharge confined to the points of suture penetration). 2. Infection of an episiotomy or newborn circumcision site. 3. Infected burn wound. 4. Incisional SSI that extends into the fascial and muscle layers (see deep incisional SSI).   **Deep incisional SSI** - Infection occurs within 30 days after the operation if no implant† is left in place or within 1 year if implant is in place and the infection appears to be related to the operation and infection involves deep soft tissues (e.g., fascial and muscle layers) of the incision and at least one of the following:   1. Purulent drainage from the deep incision but not from the organ/space component of the surgical site. 2. A deep incision spontaneously dehisces or is deliberately opened by a surgeon when the patient has at least one of the following signs or symptoms: fever (>38º C), localized pain, or tenderness, unless site is culture-negative. 3. An abscess or other evidence of infection involving the deep incision is found on direct examination, during reoperation, or by histopathologic or radiologic examination. 4. Diagnosis of a deep incisional SSI by a surgeon or attending physician.   Notes:   1. Report infection that involves both superficial and deep incision sites as deep incisional SSI. 2. Report an organ/space SSI that drains through the incision as a deep incisional SSI. |
| --- |

†National Nosocomial Infection Surveillance definition: a nonhuman-derived implantable foreign body (e.g., prosthetic heart valve, nonhuman vascular graft, mechanical heart, or hip prosthesis) that is permanently placed in a patient during surgery.
