# Supplementary material for: Management of superficial and deep surgical site infection: an international multidisciplinary consensus
Source: Updates Surg. 2021 Mar 26;73(4):1315–25. doi: 10.1007/s13304-021-01029-z (PMC8397635; doi:10.1007/s13304-021-01029-z)
Supplement: Supplementary file 3 — Supplementary file3 (DOCX 24 kb) [file 13304_2021_1029_MOESM3_ESM.docx]

**Additional file 2.** Criteria for Defining Surgical Site Infections (SSIs) based on the CDC definitions for superficial and deep incisional infections, excluding organ and body space infections.^1^

| **Superficial Incisional SSI** - Infection occurs within 30 days after the operation and infection involves only skin or subcutaneous tissue of the incision and at least one of the following:   1. Purulent drainage, with or without laboratory confirmation, from the superficial incision. 2. Organisms isolated from an aseptically obtained culture of fluid or tissue from the superficial incision. 3. At least one of the following signs or symptoms of infection: pain or tenderness, localized swelling, redness, or heat and superficial incision is deliberately opened by surgeon, unless incision is culture-negative. 4. Diagnosis of superficial incisional SSI by the surgeon or attending physician.   Do not report the following conditions as SSI:   1. Stitch abscess (minimal inflammation and discharge confined to the points of suture penetration). 2. Infection of an episiotomy or newborn circumcision site. 3. Infected burn wound. 4. Incisional SSI that extends into the fascial and muscle layers (see deep incisional SSI).   **Deep incisional SSI** - Infection occurs within 30 days after the operation if no implant† is left in place or within 1 year if implant is in place and the infection appears to be related to the operation and infection involves deep soft tissues (e.g., fascial and muscle layers) of the incision and at least one of the following:   1. Purulent drainage from the deep incision but not from the organ/space component of the surgical site. 2. A deep incision spontaneously dehisces or is deliberately opened by a surgeon when the patient has at least one of the following signs or symptoms: fever (>38º C), localized pain, or tenderness, unless site is culture-negative. 3. An abscess or other evidence of infection involving the deep incision is found on direct examination, during reoperation, or by histopathologic or radiologic examination. 4. Diagnosis of a deep incisional SSI by a surgeon or attending physician.   Notes:   1. Report infection that involves both superficial and deep incision sites as deep incisional SSI. 2. Report an organ/space SSI that drains through the incision as a deep incisional SSI. |
| --- |

†National Nosocomial Infection Surveillance definition: a nonhuman-derived implantable foreign body (e.g., prosthetic heart valve, nonhuman vascular graft, mechanical heart, or hip prosthesis) that is permanently placed in a patient during surgery.

1. Mangram AJ, Horan TC, Pearson ML, Silver LC, Jarvis WR. Guideline for Prevention of Surgical Site Infection, 1999. Centers for Disease Control and Prevention (CDC) Hospital Infection Control Practices Advisory Committee. Am J Infect Control. 1999;27:97-132.
